# Multi-mode Hybrid Plasmonic Waveguides with Enhanced Confinement and Propagation

**DOI:** 10.1007/s11468-015-0107-z

**Published:** 2015-10-10

**Authors:** John Colanduoni, Daniel Nikolov, Huizhong Xu

**Affiliations:** Department of Physics, College of Liberal Arts and Sciences, St. John’s University, 8000 Utopia Parkway, Jamaica, NY 11439 USA; Department of Physics & Astronomy, University of Southern California, 825 Bloom Walk, Los Angeles, CA 90089 USA; Institute of Optics, University of Rochester, 275 Hutchison Rd., Rochester, NY 14627 USA

**Keywords:** Subwavelength aperture, Surface plasmons, Hybrid waveguide, Extraordinary transmission

## Abstract

A hybrid waveguide, which consists of a dielectric wire above a dielectric-metal interface, has been previously proposed to achieve high confinement with low loss. By exciting this geometry with an aperture in the metal that takes advantage of the extraordinary transmission through subwavelength apertures, it is possible to strongly couple to multiple modes. The real part of the fundamental mode is in fact capable of exceeding the index of refraction of all the materials used while maintaining a manageable imaginary part, as a result of appropriate choice of materials for the dielectric wire and the metal. In addition, as the confinement of the second mode is comparable to that of the fundamental mode but has a much longer propagation length, this mode can be utilized in light-guiding applications where enhanced confinement and propagation is desired.

## Introduction

Waveguides capable of achieving high confinement with low loss are a key goal in the developing field of plasmonics, with many significant applications on the horizon [1–9]. Plasmonic circuits promise improved photonic devices that will be able to compete with current electronic semiconductor technology [10]. Energy-efficient and compact photonic circuits would revolutionize telecommunications technology with all-optical switches that would drastically increase the throughput and efficiency of existing optical networks [11]. Plasmonic metamaterials have significant promise in the development of superlenses that operate in the visible range [12, 13], allowing for unprecedented resolution that would expand our ability to examine nanoscale aspects of various physical processes. Molecular sensors that use surface plasmons have also been experimentally realized, opening up new possibilities in biosensing [14]. Plasmonic effects have also found utility in the development of high-efficiency photovoltaic devices [15, 16]. In addition, subwavelength lithography that takes advantage of surface plasmon polariton resonance has been demonstrated [17].

A hybrid waveguide with excellent confinement and propagation was recently proposed by Oulton et al. [18]. This waveguide geometry consists of a dielectric-metal interface between SiO_2_ and Ag, with a GaAs cylinder placed above the interface, inside the SiO_2_ half. Their focus was on light at telecommunications wavelength (1550 nm). Their simulations show that using this configuration, this waveguide supports a single hybrid mode which can be well modeled by a coupled mode theory of the individual modes of the cylinder and the metal-dielectric interface.

## The Model

**Fig. 1:**
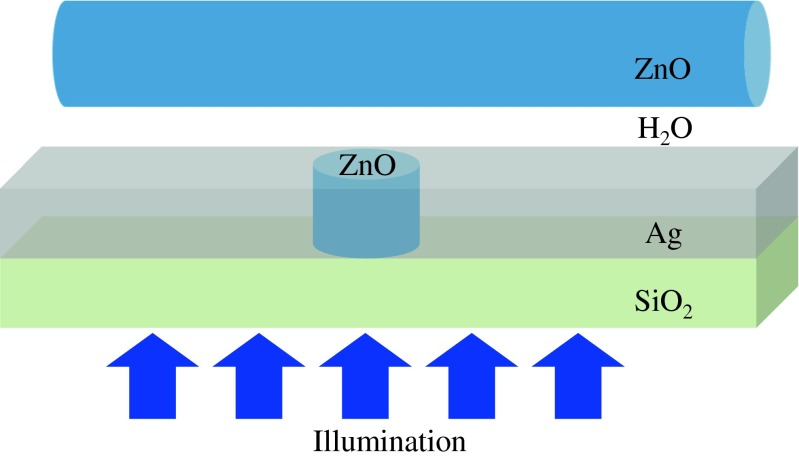
Multi-mode hybrid waveguide model

In this study, we focus on combining this hybrid waveguide geometry with the configuration of extraordinary transmission through subwavelength dielectric apertures examined previously [19, 20] to form a composite waveguide enabling enhanced confinement and propagation properties. This new waveguide geometry is composed of a horizontal cylindrical nanowire waveguide above a metal film of fixed thickness (100 nm in our work) and a vertical 40-nm-diameter cylindrical aperture in the metal which is made of the same material as the horizontal waveguide and placed directly underneath it, as shown in Fig. 1. Excitation is provided by coherent, linearly polarized illumination of the aperture from the substrate below. As the transmission through the aperture requires materials with properly matched dielectric constants, the wavelength of the incident light is chosen to be 488 nm and the materials for the metal, first, and second dielectrics are chosen to be silver, water, and zinc oxide (ZnO), respectively. The medium below the aperture is taken to be SiO_2_.

The changes in geometry and the material properties result in significantly different behavior of this new waveguide. The first difference is that multiple modes are always present in this waveguide. The surface plasmon mode, which originates from excitation characteristic of the aperture, can be well described by a Hankel function of the first kind with wavenumber equal to the characteristic surface plasmon wavenumber of the H_2_O/Ag interface [21]. This mode, although important near the aperture, falls off quickly along the axis of the horizontal cylinder. The remaining modes are sinusoidal along the horizontal cylinder. For cylinder diameters above 120 nm, these modes can be found in two-dimensional (2D) eigenvalue simulations (COMSOL) of the cross section of the horizontal cylinder/dielectric/metal interface, although many of the possible 2D modes may have zero/negligible amplitude under the excitation provided by the aperture. For diameters below 120 nm, exotic modes that are not found in 2D simulations also appear as a result of the unusual form of excitation. The 2D simulations show that the first two sinusoidal modes display subwavelength confinement and that the second mode has a slightly better confinement and a much longer propagation distance than the fundamental mode. As shown in the three-dimensional (3D) simulations (COMSOL), the fundamental mode has a higher amplitude, but the second mode starts to dominate the fields further away from the aperture as a result of its slow attenuation making it very attractive in confined light guiding applications.

## Results and Discussion

### 2D Analysis

**Fig. 2:**
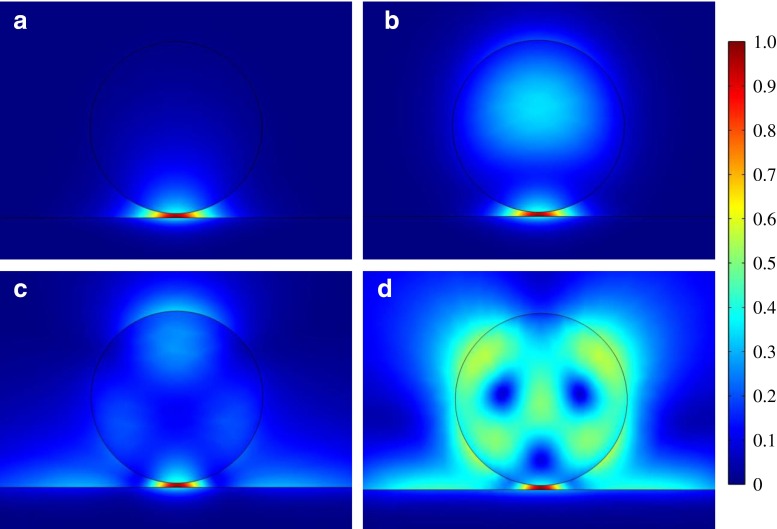
Distribution of |*E*| across the cross-sectional area of the waveguide for **a** fundamental mode, **b** second mode, **c** third mode, and **d** fourth mode. The maximum value of |*E*| is chosen to be 1 V/m for all modes. The gap size is 10 nm and the diameter of the horizontal waveguide is 400 nm

Two-dimensional eigenvalue simulations of the horizontal cylinder/metal interface geometry studied by Oulton et al. with our new choice of materials and wavelength show some new properties, even before the excitation source is introduced. The first few modes that exhibit strong confinement in the gap fall into one of four characters, as shown in Fig. 2. The fundamental mode is always the mode of first character, and the rest of the characters generally have successively lower mode indices. Depending on the gap size and cylinder radius, not all these modes will be present; in particular, the third and fourth modes only show up for very high diameters, as one would expect. The modes disappear when their indices approach that of water (1.34).

**Fig. 3:**
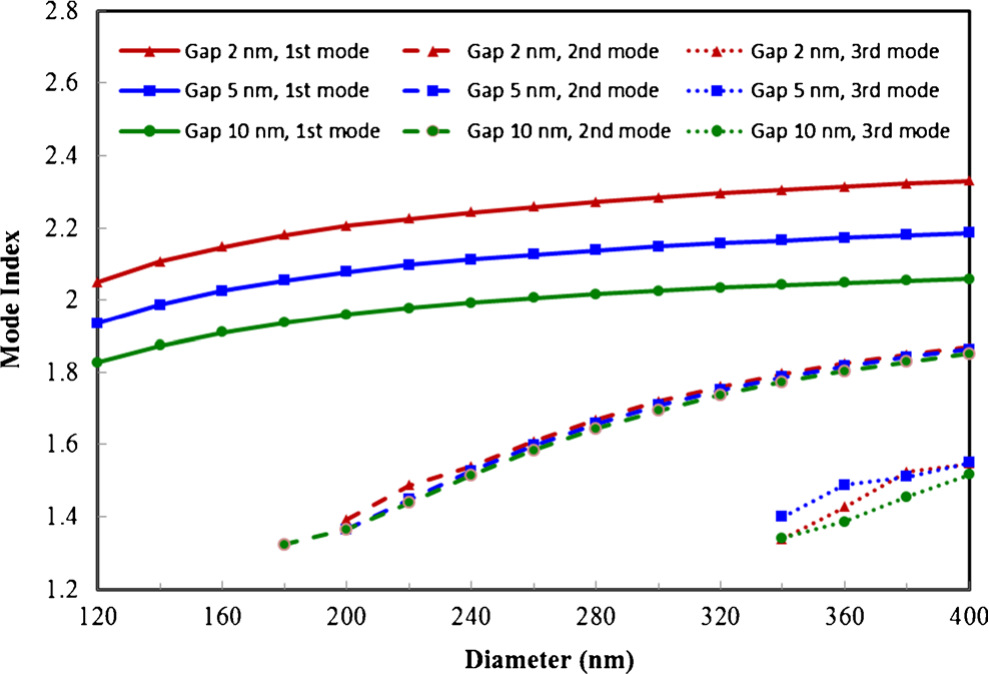
Effective mode indices of fundamental mode (*solid lines*), second mode (*dashed lines*), and third mode (*dotted lines*) as a function of the horizontal waveguide diameter for three different gap sizes: 2, 5, and 10 nm

The effective indices of the modes are shown in Fig. 3. We see that the real part of the effective index of the fundamental mode levels off as the diameter increases. For the 10 nm gap waveguides, the limiting value is around 2.06, the refractive index of ZnO for 488 nm light [22], as expected. However, for the 5 nm and 2 nm gap waveguides, the mode indices surpass the refractive index of ZnO by a considerable amount. This can be understood as the result of matching the dielectric constant of Ag, which is taken to be −7.879 + 0.736*i* [23], with that of ZnO on the cylinder/dielectric/metal interface:

1 $$ {\varepsilon}_{\mathrm{Ag}}+{\varepsilon}_{\mathrm{ZnO}}\approx 0. $$

We note that the above equation is the exact condition for the resonant excitation of surface plasmon polaritons at the planar metal dielectric interface [24]. For the hybrid modes, strong interaction is expected between the bottom surface of the cylinder and the metal surface as manifested by the enhancement of fields inside the gap. As the gap size decreases and the diameter of the horizontal cylinder increases, the cylinder/dielectric/metal interface behaves more like a planar ZnO/Ag interface and surface plasmon polaritons are resonantly excited as a result of the matching of their dielectric constants. As discussed below, simulations using a fictitious metal which satisfies Eq. (1) more closely show that the effective index of the fundamental mode can be much greater than that of ZnO and exhibit even better confinement. This demonstrates that material matching is in fact what produces the high confinement in this waveguide geometry.

**Fig. 4:**
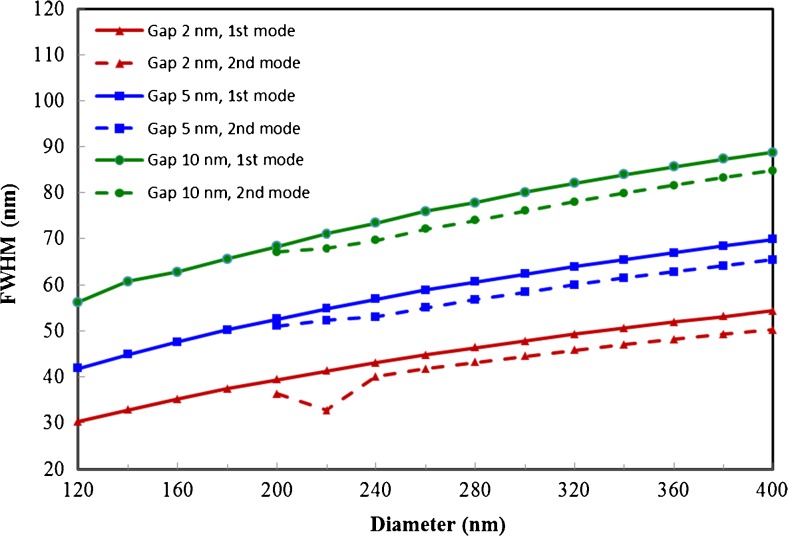
Lateral width of fundamental mode (*solid lines*) and second mode (*dashed lines*) in the middle of the gap as a function of the horizontal waveguide diameter for three different gap sizes: 2, 5, and 10 nm

**Fig. 5:**
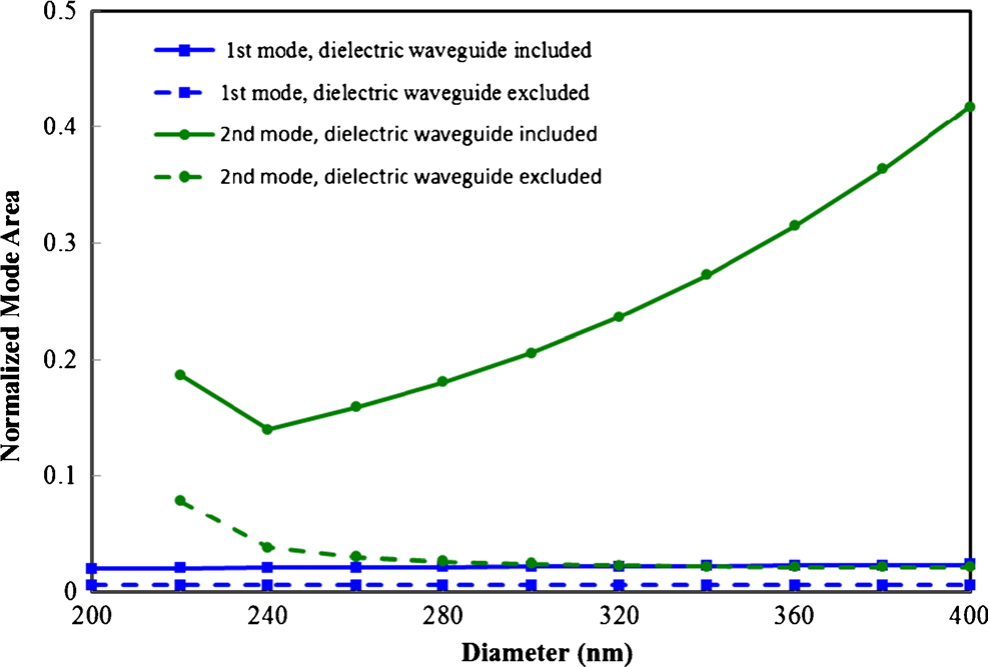
Effective mode area normalized by the diffraction limit *λ* ^2^/4 as a function of the horizontal waveguide diameter for fundamental mode (*squares*) and second mode (*circles*). The gap size is 2 nm. To calculate the effective mode area, the total energy density is first integrated over the cross-sectional area of the waveguide then divided by the maximum energy density. The calculation is done with the contribution of the fields inside the dielectric waveguide included (*solid lines*) and excluded (*dashed lines*)

As shown in Fig. 4, the modes of the first and second character have good confinement within the gap, and smaller gap sizes/diameters result in improved confinement as can be seen from the full width half maximum (FWHM) of the field distribution along the lateral direction in the middle of the gap. It can also be seen that the second mode seems to have a slightly smaller FWHM than the fundamental mode. To better characterize the confinement, we have adopted the same method used in ref. [18] to calculate the effective mode area (see Appendix A). The total energy density is first integrated over the cross-sectional area of the waveguide then divided by the maximum energy density to obtain the effective mode area, which is then normalized by the diffraction limit *λ*^2^/4. The results are shown as solid lines in Fig. 5. Clearly, the second mode has much greater effective mode area than the fundamental mode due to the fields inside the dielectric waveguide. However, when one excludes the contribution of the fields inside the dielectric waveguide in calculating the effective mode area (see dashed line in Fig. 5), a significant reduction in the effective mode area is observed. For a gap size of 2 nm and a 400-nm-diameter waveguide, we found this effective mode area for the second mode is only 1/50 of the diffraction limit *λ*^2^/4, making this mode useful for certain applications where molecules do not exist inside the dielectric waveguide, thus, cannot be excited by the fields inside the dielectric waveguide.

**Fig. 6:**
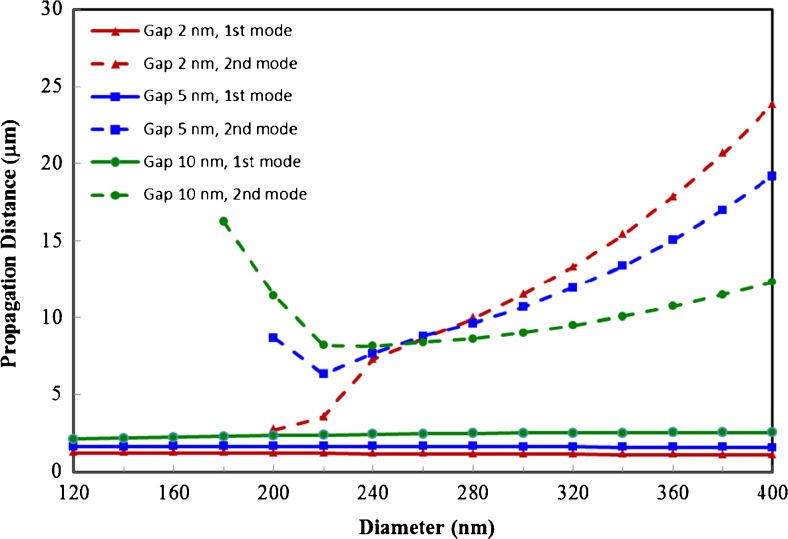
Propagation distance of fundamental mode (*solid lines*) and second mode (*dashed lines*) as a function of the horizontal waveguide diameter for three different gap sizes: 2, 5, and 10 nm

As can be seen in Fig. 6, the fundamental mode displays the expected drop in propagation distance, defined as the distance before the amplitude of the mode drops by a factor of 1/*e*, for smaller gap sizes and diameters (i.e., higher confinement). The second mode, on the other hand, exhibits increased propagation length for certain reduced gap size and diameter combinations. In addition, the second mode has the advantage that its propagation distance is always much higher than that of the fundamental mode.

### 3D Analysis

Although the 2D simulations show that the four mode characters described above can be supported by the hybrid waveguide with these materials, it does not demonstrate that the higher order modes are actually accessible. However, we found that this can be achieved by exciting the horizontal waveguide through a subwavelength ZnO nanowire that is situated directly below the horizontal waveguide and embedded inside the silver film. We performed 3D simulations of the entire waveguide geometry with perfectly matched layer (PML) boundary conditions [19] to establish the multi-mode nature of this combined geometry.

**Fig. 7:**
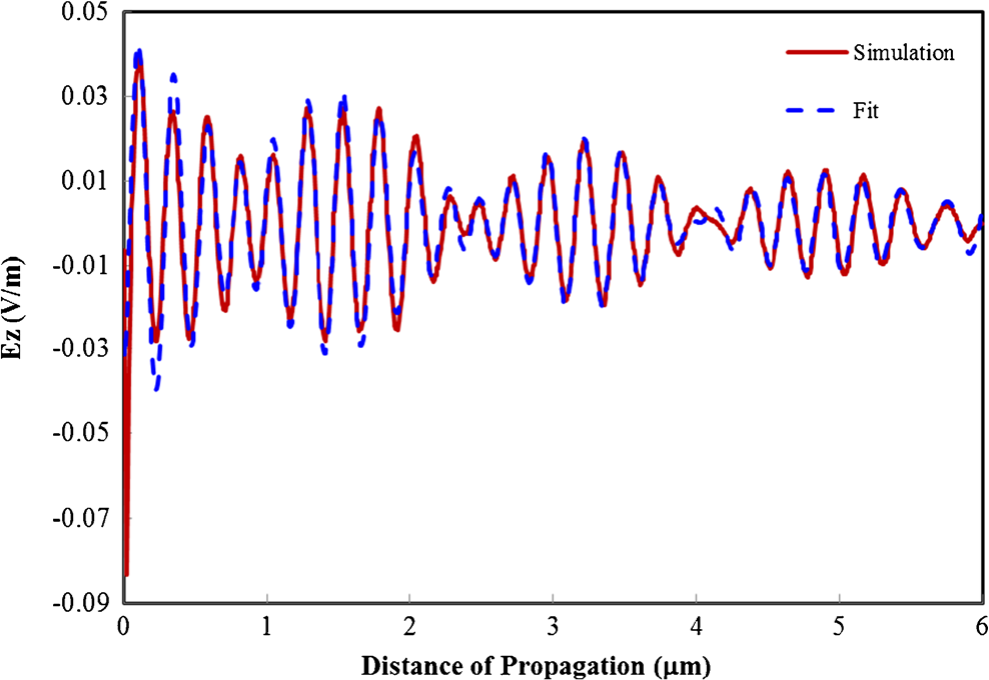
The *z*-component of the electric field from 3D simulation compared to the fit based on a superposition of the cylindrical surface plasmon mode and the sinusoid modes. The gap size is 10 nm and the diameter of the horizontal waveguide is 320 nm

**Fig. 8:**
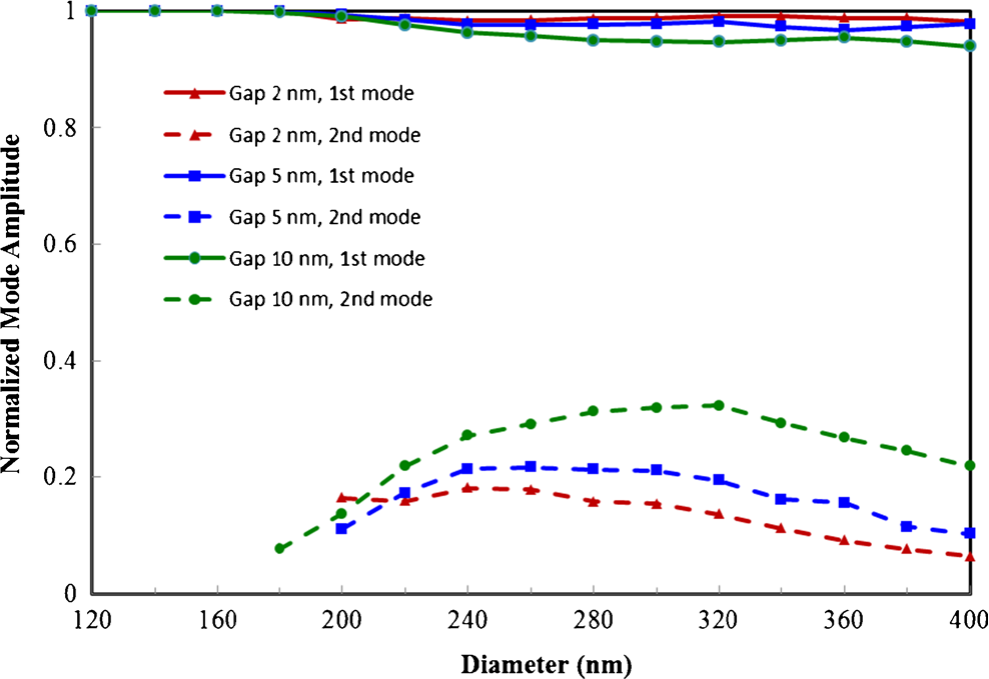
Normalized mode amplitudes of fundamental mode (*solid lines*) and second mode (*dashed lines*) as a function of the horizontal waveguide diameter for three different gap sizes: 2, 5, and 10 nm

We found that in cases where the coupling between the waveguides was not too strong (i.e., the horizontal cylinder diameter was above 120 nm), the field along the horizontal waveguide was well represented as a superposition of the cylindrical surface plasmon mode characteristic of the aperture and the modes found in the 2D simulations (see Appendix B). This is demonstrated by an example shown in Fig. 7 for a gap size of 10 nm and a diameter of 320 nm for the horizontal waveguide. This allows us to successfully model the confinement of the complete 3D geometry, even as the cylindrical surface plasmon mode falls off very quickly as we move away from the aperture. As can be seen from the normalized mode amplitudes in Fig. 8, the auxiliary modes have significant amplitudes demonstrating that the desirable second mode is in fact accessible. Due to the short propagation distance but high amplitude of the fundamental mode, it dominates the fields near the aperture but is overshadowed by the second mode as we move further from the excitation. Due to the good confinement of both modes, decent field strength with a confinement of ∼40 nm can be obtained at a distance of 40*λ* from the excitation aperture.

### Improved Matching

We attribute the high confinement and high effective indices for this new waveguide geometry to the material matching condition mentioned previously. As shown in the previous analysis of extraordinary transmission through a single isolated aperture [20], the matching condition leads to a diminishment of the in-plane wavenumber and a weak dependence on the in-plane radial coordinate, a characteristic feature of the plasmonic mode. This is also the case here, as the exceptionally high out-of-plane index leads to a very small in-plane index.

**Fig. 9:**
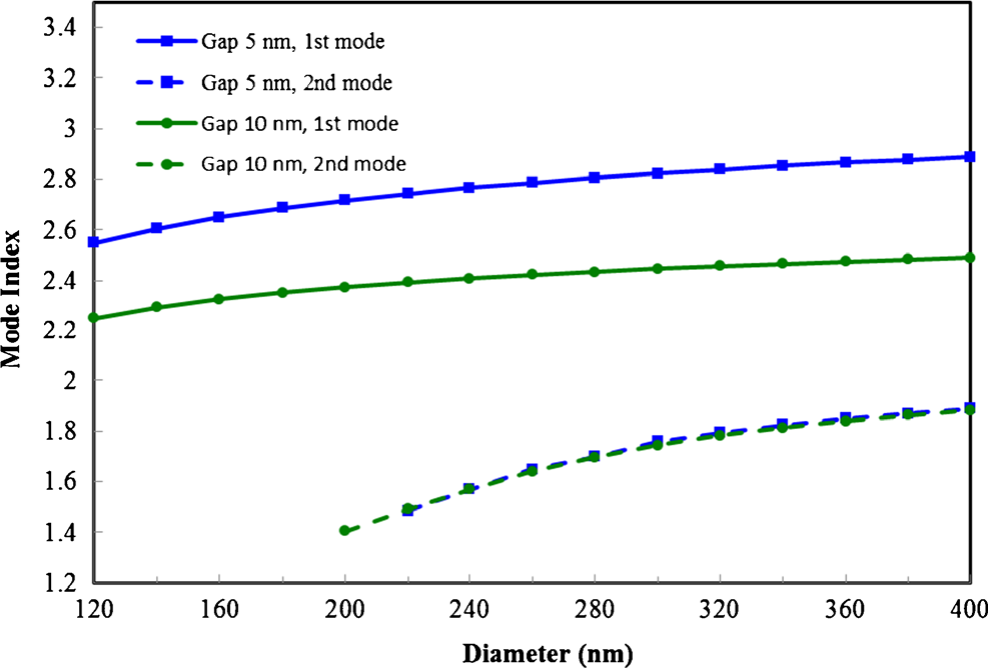
Effective mode indices of fundamental mode (*solid lines*) and second mode (*dashed lines*) with improved material matching as a function of the horizontal waveguide diameter for two different gap sizes: 5 and 10 nm

**Fig. 10:**
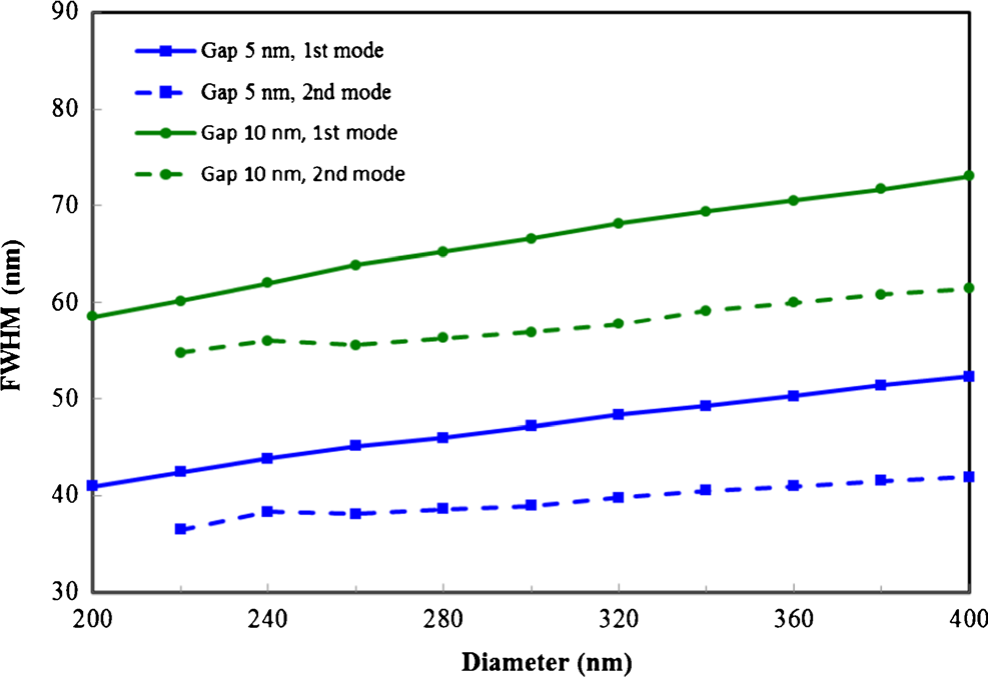
Lateral width of fundamental mode (*solid lines*) and second mode (*dashed lines*) with improved material matching as a function of the horizontal waveguide diameter for two different gap sizes: 5 and 10 nm

To demonstrate the effect of material matching, we performed simulations using the same geometry but with modified materials that satisfy Eq. (1) better. All refractive indices were kept the same except for that of the metal (Ag), which was changed to 0.0963–2.0659*i*. As shown in Figs. 9 and 10, this change results in even higher mode indices and improved confinement. However, when the new refractive index is used, additional modes other than the ones found in 2D simulations need to be taken into consideration for all cylinder diameters with the 2 nm gap. This shows that the material matching condition greatly increases the coupling between the horizontal and vertical cylinders, which can result in a radical change in the behavior of this geometry that bears further investigation.

## Conclusion

We have examined a novel plasmonic waveguide geometry for use in applications requiring high confinement and low attenuation. We have shown that modes with high confinement can be obtained by taking advantage of the same material matching condition that makes extraordinary transmission through a subwavelength aperture possible. In addition, the unique nature of our composite waveguide geometry allows us to access high-order modes. This is essential to the operation of this waveguide, as the second mode shows more desirable and exceptional characteristics, including longer propagation length, superior confinement, and an actual increase in the propagation length as confinement improves in certain configurations.

## Appendix A: Definition of Mode Confinement and Mode Area

To find the modes, a 2D eigenvalue simulation of the vertical cross section of the horizontal waveguide (without the vertical cylinder) is performed. This results in a set of modes, defined by their corresponding transverse field distributions and out-of-plane mode indices. Confinement of the modes is determined by the full width half maximum (FWHM) of the field distribution along a line through the middle of the gap between the cylinder and the metal and perpendicular to the axis of the cylinder.

The mode area is calculated by integrating the total energy density $W\left(\overrightarrow{r}\right)$ over the cross-sectional area of the waveguide and dividing it by the maximum energy density:

2 $$ {A}_m=\frac{{\displaystyle \iint W\left(\overrightarrow{r}\right){d}^2\overrightarrow{r}}}{W_0}, $$

where *W*_0_ is the maximum energy density. In certain applications where the fields inside the dielectric waveguide cannot be used to excite molecules, it is useful to calculate the mode area by integrating the total energy density $W\left(\overrightarrow{r}\right)$ over the cross-sectional area outside the dielectric waveguide:

3 $$ {A}_m^{\prime }=\frac{{\displaystyle {\iint}_{r>a}W\left(\overrightarrow{r}\right){d}^2\overrightarrow{r}}}{W_0}, $$

where *a* is the radius of the cylindrical waveguide. Both mode areas for each mode are then normalized by the diffraction limit *λ*^2^/4 and plotted in Fig. 5.

## Appendix B: Calculation of Mode Amplitude

The fitting of the 3D simulation results is obtained by analyzing *E*_*z*_, the component of the electric field perpendicular to the metal dielectric interface, along the line that is parallel to the axis of the horizontal cylinder in the middle of the gap between the metal and the cylinder. The field from the simulations is fitted using the following function:

4 $$ {E}_z=A{H}_1^{(1)}\left({k}_{\mathrm{SPP}}x\right)+{\displaystyle \sum_j{B}_j \exp \left(i{k}_0{n}_jx\right)}, $$

where *k*_SPP_ is the propagation constant of the surface plasmon polaritons (SPPs) at the water/silver interface, *k*_0_ is the free space wavenumber, *n*_*j*_ is the mode index of the *j*th sinusoidal mode (as obtained from the 2D simulations), and *A* and *B*_*j*_ are complex amplitudes for the SPP mode and the *j*th sinusoidal mode, respectively. The fit is performed using complex linear least squares, producing values for *A* and *B*_*j*_. Initially, the mode indices of all modes from the 2D eigenvalue simulation are included in the summation, but the amplitudes of most higher order modes are negligible. The normalized amplitudes are then calculated for each sinusoidal mode by dividing *B*_*j*_ by the square root of the sum of the squares of the absolute values of the amplitudes.
